# Artificial intelligence in health care: accountability and safety

**DOI:** 10.2471/BLT.19.237487

**Published:** 2020-02-25

**Authors:** Ibrahim Habli, Tom Lawton, Zoe Porter

**Affiliations:** a Department of Computer Science, University of York, Deramore Lane, Heslington, York YO10 5GH, England.; b Bradford Teaching Hospitals NHS Foundation Trust, Bradford, England.; c Department of Philosophy, University of York, York, England.

## Abstract

The prospect of patient harm caused by the decisions made by an artificial intelligence-based clinical tool is something to which current practices of accountability and safety worldwide have not yet adjusted. We focus on two aspects of clinical artificial intelligence used for decision-making: moral accountability for harm to patients; and safety assurance to protect patients against such harm. Artificial intelligence-based tools are challenging the standard clinical practices of assigning blame and assuring safety. Human clinicians and safety engineers have weaker control over the decisions reached by artificial intelligence systems and less knowledge and understanding of precisely how the artificial intelligence systems reach their decisions. We illustrate this analysis by applying it to an example of an artificial intelligence-based system developed for use in the treatment of sepsis. The paper ends with practical suggestions for ways forward to mitigate these concerns. We argue for a need to include artificial intelligence developers and systems safety engineers in our assessments of moral accountability for patient harm. Meanwhile, none of the actors in the model robustly fulfil the traditional conditions of moral accountability for the decisions of an artificial intelligence system. We should therefore update our conceptions of moral accountability in this context. We also need to move from a static to a dynamic model of assurance, accepting that considerations of safety are not fully resolvable during the design of the artificial intelligence system before the system has been deployed.

## Résumé

La perspective que les décisions prises par un outil clinique basé sur l'intelligence artificielle puissent porter préjudice aux patients est un concept dont les bonnes pratiques de responsabilité et de sécurité actuelles ne tiennent pas encore compte à travers le monde. Nous nous concentrons sur deux aspects qui caractérisent les décisions de l'intelligence artificielle à usage clinique : la responsabilité morale des préjudices aux patients, et la garantie de sécurité pour protéger les patients contre de tels préjudices. Les outils fondés sur l'intelligence artificielle remettent en cause les pratiques cliniques conventionnelles d'attribution des responsabilités et de garantie de la sécurité. Les décisions formulées par les systèmes d'intelligence artificielle sont de moins en moins soumises au contrôle des médecins et spécialistes de la sécurité, qui ne comprennent et ne maîtrisent pas toujours les subtilités régissant cette prise de décision. Nous illustrons notre analyse en l'appliquant à un exemple de système d'intelligence artificielle développé dans le cadre du traitement des infections. Le présent document se termine par une série de suggestions concrètes servant à identifier de nouveaux moyens de tempérer ces inquiétudes. Nous estimons qu'il est nécessaire d'inclure les développeurs à l'origine de l'intelligence artificielle ainsi que les spécialistes de la sécurité des systèmes dans notre évaluation de la responsabilité morale des préjudices causés aux patients. Car pour l'instant, aucun des acteurs impliqués dans le modèle ne remplit pleinement les conditions traditionnelles de responsabilité morale pour les décisions prises par un dispositif d'intelligence artificielle. Dans ce contexte, il est donc essentiel revoir notre conception de la responsabilité morale. Nous devons également passer d'un modèle de garantie statique à un modèle de garantie dynamique, et accepter que certains impératifs de sécurité ne puissent être entièrement résolus durant l'élaboration du système d'intelligence artificielle, avant sa mise en œuvre.

## Resumen

La perspectiva de que los pacientes sufran daños a causa de por las decisiones tomadas por un instrumento clínico de inteligencia artificial es un aspecto al que todavía no se han ajustado las prácticas actuales de responsabilidad y seguridad en todo el mundo. El presente documento se centra en dos aspectos de la inteligencia artificial clínica utilizada para la toma de decisiones: la responsabilidad moral por el daño causado a los pacientes y la garantía de seguridad para proteger a los pacientes contra dicho daño. Las herramientas de inteligencia artificial están desafiando las prácticas clínicas estándar de asignación de responsabilidades y de garantía de seguridad. Los médicos clínicos y los ingenieros de seguridad de las personas tienen menos control sobre las decisiones que adoptan por los sistemas de inteligencia artificial y menos conocimiento y comprensión de la forma precisa en que los sistemas de inteligencia artificial adoptan sus decisiones. Este análisis se ilustra aplicándolo a un ejemplo de un sistema de inteligencia artificial desarrollado para su uso en el tratamiento de la sepsis. El documento termina con sugerencias prácticas sobre las vías de acción para mitigar estas preocupaciones. Se sostiene la necesidad de incluir a los desarrolladores de inteligencia artificial y a los ingenieros de seguridad de sistemas en las evaluaciones de la responsabilidad moral por los daños causados a los pacientes. Entretanto, ninguno de los actores del modelo cumple sólidamente las condiciones tradicionales de responsabilidad moral por las decisiones de un sistema de inteligencia artificial. En consecuencia, se debería actualizar nuestra concepción de la responsabilidad moral en este contexto. También es preciso pasar de un modelo de garantía estático a uno dinámico, aceptando que las consideraciones de seguridad no se pueden resolver plenamente durante el diseño del sistema de inteligencia artificial antes de que el sistema sea implementado.

## ملخص

إن احتمال حدوث ضرر للمريض نتيجة للقرارات التي يتم اتخاذها بواسطة أداة سريرية تعتمد على الذكاء الاصطناعي، هو أمر لم يتم بعد ضبط ممارسات المساءلة والسلامة بالنسبة له في جميع أنحاء العالم. نحن نقوم بالتركيز على جانبين من الذكاء الاصطناعي السريري يتم استخدامهما لصنع القرار: المساءلة الأخلاقية عن الضرر الذي يلحق بالمرضى؛ وضمان السلامة لحماية المرضى من هذا الضرر. تتحدى الأدوات التي تعتمد على الذكاء الاصطناعي الممارسات السريرية القياسية الخاصة بتوجيه اللوم وضمان السلامة. الأطباء البشريون ومهندسو السلامة لديهم سيطرة أضعف على القرارات التي توصلت إليها أنظمة الذكاء الاصطناعي، كما أن لديهم معرفة وفهم أقل لكيفية وصول أنظمة الذكاء الاصطناعي بشكل محدد إلى هذه القرارات. نقوم بتوضيح هذا التحليل من خلال تطبيقه على مثال لنظام يعتمد على الذكاء الاصطناعي، تم تطويره للاستخدام في علاج الإنتان. يوجد بخاتمة الورقة اقتراحات عملية لطرق المضي قدما لتخفيف هذه المخاوف. نحن ندافع عن الحاجة لتضمين مطوري الذكاء الاصطناعي، ومهندسي سلامة النظم، في تقييماتنا للمساءلة الأخلاقية تجاه الأذى الذي يلحق بالمريض. وفي نفس الوقت، لا تفي أي من الجهات الفاعلة في هذا النموذج، بشكل فعال بالشروط التقليدية للمساءلة الأخلاقية عن القرارات التي يتخذها نظام الذكاء الاصطناعي. وبالتالي، يجب علينا تحديث ما لدينا من مفاهيم بشأن المساءلة الأخلاقية في هذا السياق. كما نحتاج كذلك إلى الانتقال من نموذج استاتيكي إلى نموذج ديناميكي للتأكيد، وقبول أن هذه الاعتبارات الخاصة بالسلامة ليست قابلة للحل بشكل كامل أثناء تصميم نظام الذكاء الاصطناعي، قبل نشر هذا النظام.

## 摘要

基于人工智能的临床工具所做的决定可能会对患者造成伤害，而目前世界范围内的问责和安全实践尚未对此作出调整。我们重点关注人工智能制定临床决策的两个方面问题：对患者造成伤害的道德问责；以及安全确保保护患者免受此类伤害。基于人工智能的工具正在挑战追究责任和确保安全的标准临床实践。临床医生和安全工程师对人工智能系统做出的决策的控制力较弱，在人工智能系统如何准确地做出决策方面的知识和理解水平也较低。我们通过一个用于治疗败血症的人工智能系统作为示例，来阐述这一分析。文章最后对如何缓解这些担忧提出了切实可行的建议。我们认为十分有必要让人工智能开发人员和系统安全工程师参与我们对患者伤害的道德问责评估中。同时，模型中没有任何参与者可以很好地满足人工智能系统决策中道德问责的传统条件。因此，我们应该在这方面更新我们的道德问责观念。我们还需要从静态的保障模式转变为动态的保障模式，尽管部署人工智能系统之前，人工智能系统设计过程中安全方面的顾虑尚未完全解决。

## Резюме

Вероятность причинения вреда пациентам в результате принятия решений с помощью клинических инструментов, основанных на использовании искусственного интеллекта, пока что не учитывается в нынешних мировых практиках обеспечения прозрачности и безопасности. Авторы уделяют особое внимание двум аспектам применения искусственного интеллекта в клинической практике для принятия решений: моральной ответственности за вред, причиненный пациентам, и обеспечению безопасности для защиты пациентов от такого вреда. Инструменты, основанные на использовании искусственного интеллекта, бросают вызов стандартной клинической практике распределения ответственности и обеспечения безопасности. Лечащие врачи и инженеры по технике безопасности имеют небольшое влияние на решения, принимаемые с использованием систем искусственного интеллекта, и не обладают полными знаниями и пониманием тонкостей процесса принятия решений системами искусственного интеллекта. С целью наглядной демонстрации такого анализа авторы приводят пример системы на основе искусственного интеллекта, разработанной для использования при лечении сепсиса. В конце документа приводятся практические предложения по решению этих проблем. Авторы настаивают на необходимости включения разработчиков искусственного интеллекта и инженеров систем безопасности в процесс оценки моральной ответственности за вред, причиненный пациенту. Между тем ни один из участников модели полностью не удовлетворяет традиционным условиям, предъявляемым к моральной ответственности за решения, принимаемые системой искусственного интеллекта. В связи с этим необходимо пересмотреть понятие моральной ответственности в данном контексте. Требуется также перейти от статической к динамической модели обеспечения гарантий, признав, что невозможно полностью учесть соображения безопасности при разработке системы искусственного интеллекта до ее развертывания.

## Introduction

Recent research has demonstrated the potential to create artificial intelligence-based health-care applications that can reach or exceed the performance of clinicians for specific tasks.^1^ These applications could help to address major global challenges, including shortages of clinicians to meet the demands of ageing populations and the inequalities in access to health care in low-resource countries. Health care, however, is a complex, safety-critical domain in which technological failures can lead directly to patient harm.^2^

The prospect of patient harm caused by the decisions made by an artificial intelligence-based clinical tool is something to which current practices of moral accountability and safety assurance worldwide have not yet adjusted. In this paper we focus on two implications of clinical decision-making that involves artificial intelligence: moral accountability for harm to patients; and safety assurance to protect patients against such harm. Our central thesis is that digital tools are challenging the standard clinical practices of assigning blame, as well of assuring safety. We use an example from an artificial intelligence-based clinical system developed for use in the treatment of sepsis. We discuss this system’s perceived and actual benefits and harms, and consider the moral accountability and safety assurance issues that arise from the perspective of both clinicians and patients. We conclude with practical suggestions for dealing with moral accountability and safety assurance in the use of artificial intelligence in health care.

### Moral accountability

Moral responsibility concerns, among other things, accountability for one’s decisions and actions.^3^ We will use the terms moral responsibility and moral accountability interchangeably. It is important, however, to distinguish moral accountability from legal liability. Though closely related, the former can exist in the absence of the latter, and vice versa. We do not consider questions of law in this paper. 

In the past 50 years there has been a strong trend in philosophy to think that making moral responsibility judgements, such as blaming and praising, is an inherently social practice.^4^ These judgements express reactions such as resentment or gratitude for how we have been treated by others and whether this treatment corresponds to our interpersonal expectations and demands.^4^ Many philosophers define two conditions for a person to be morally responsible for an action: the control condition (control over the decision or action, where loss of control is not due to recklessness) and the epistemic condition (sufficient understanding of the decision or action and its likely consequences, where ignorance is not due to negligence).^5^^,^^6^ These conditions can be traced back to the writings of the Greek philosopher Aristotle in the 4th century BCE.^7^ Failure to meet these conditions would excuse a person from moral responsibility.

Numerous academic philosophers have written about what constitutes relevant control or sufficient understanding in the context of moral responsibility.^8^ Nonetheless, when artificial intelligence systems are involved in the decision-making process, it is uncertain how far it would be reasonable to hold human clinicians accountable for patient harm. First, clinicians do not exercise direct control over what decisions or recommendations a system reaches. Second, many artificial intelligence systems are inherently opaque,^9^ so a clinician’s understanding of precisely how the system translates input data into output decisions is difficult, if not impossible, to achieve.

Many artificial intelligence systems in health care, including the system that we describe in this paper, are assistive systems. In such cases, human clinicians make the final decision about whether to act on the system’s recommendations. In respect of this final decision or choice, human clinicians therefore meet the control and epistemic conditions mentioned earlier. However, this final choice is only half of the picture: the clinician cannot directly change the system’s internal decision-making process once it is underway, and cannot be sure that the software is reaching conclusions that reflect his or her clinical intentions. The clinician also has epistemic uncertainty about how the recommendation was reached. Furthermore, the choice to implement the recommendations will be affected by wider structural and organizational factors, such as the clinician’s workload and the development of reliance on automation.^10^ Assigning the final decision to a clinician creates moral and safety dilemmas for them, as we discuss later in the article. Delegating a part of the decision-making process to artificial intelligence systems raises important questions about how far a clinician is accountable for patient harm.

### Safety assurance

Safety assurance is concerned with demonstrating confidence in a system’s safety. Assurance of safety is commonly communicated through a safety case – a document written by the developers of the technology or service – which provides a reasoned argument supported by a body of evidence. The safety case explains why a system is acceptably safe to operate as intended in a defined environment. As emphasized by the Health Foundation,^11^

“the act of establishing and documenting a safety case helps expose existing implicit assumptions and risk acceptance judgements. Having documented a case, it becomes easier to review the arguments, question the evidence and challenge the adequacy of the approach presented. This creates greater transparency in the overall process.”

As such, the safety case helps by making an explicit statement of implicit understanding. Transparency in the design of artificial intelligence technologies, especially when the functionality is safety-critical, makes a safety case essential for health-care applications.^12^

To date, the combination of high risk of harm and strict regulation has limited the scope and authority of digital health interventions. There has therefore been only limited transfer of clinical decision-making from clinicians to digital systems. Critical software functions have been tightly defined so that the software exhibits predictable behaviour, for example in controlling infusion pumps or pacemakers or in robot-assisted surgery where the tools are under the direct control of clinical professionals. These limitations have been necessary to ensure that qualified clinicians are able to interpret dynamically complex variables related to patients and the clinical and social context. Artificial intelligence systems have shown the potential to improve clinicians’ interpretation and the subsequent decision-making process.^13^ The potential benefits of this capability, however, are offset by the widening of responsibility gaps and the additional risk of negative side-effects that are inherent in health-care interventions.^14^ In essence, the increasing of the scope and authority of digital health systems is challenging existing safety assurance practices and clinical accountability models.^15^ These safety assurance and moral accountability challenges explain some of the reluctance of safety-critical industries, such as aviation and nuclear power to consider applications of artificial intelligence.

## Example system

The example artificial intelligence system we consider here concerns the treatment of sepsis. Sepsis is “a life-threatening organ dysfunction caused by a dysregulated host response to infection”^16^ and has become a global health challenge, overtaking myocardial infarction and stroke as a cause of hospital admission, even in wealthy countries.^17^ Sepsis may progress to septic shock, where intravenous fluids alone are insufficient to maintain blood pressure and vasopressor medications are required. Patients with septic shock have a hospital mortality of over 40%, and are usually looked after in a critical care or intensive care unit.^16^ Historically, the standard treatment in critical care has been to establish a target mean arterial blood pressure (traditionally greater than 65 mmHg), administer fluids intravenously until no further improvement is seen (but usually a minimum of 30 mL/kg), and to start vasopressor medications if shock has not resolved thereafter.^18^ However, it is gradually becoming clear that a single target for blood pressure in sepsis is not appropriate, and the balance of fluids versus vasopressors to achieve it is still subject to debate.^19^

To help address the challenge of optimizing treatment, researchers at Imperial College London have developed the Artificial Intelligence (AI) Clinician,^20^ a proof-of-concept system that uses reinforcement learning to recommend actions for the care of patients fulfilling the third international consensus definitions for sepsis and septic shock.^16^ As these patients have a 90-day mortality in the region of 20%, the system’s software is trained on avoiding deaths. The system analyses 48 features from an electronic patient record and uses a Markov decision process to simulate clinical decision-making, recommending doses of fluids and vasopressors in broad bands for each 4-hour window. Thus far, the system has been developed using retrospective data and is still being evaluated off-policy (without following its advice), but the developers envisage prospective trials in the future. In the next two sections, we illustrate key moral accountability and safety assurance gaps introduced by this type of artificial intelligence-based capability.

## Potential benefits and harms

AI Clinician offers recommendations for personalized treatment based empirically on the outcomes of thousands of patients, without simply choosing an arbitrary target blood pressure, in fact, operating without any specific target at all. It has been shown that human clinicians can be distracted by competing pressures at work and are subject to clinical inertia (keeping a treatment unchanged despite changes in the patient’s clinical picture).^21^ By contrast, the digital system is ever-vigilant, providing individualized recommendations every 4 hours. It is important to note, however, that it is not clear that a physician’s inertia is always harmful in health care. The Markov decision process is a mathematical model of outcomes which are partly random and partly determined by decisions made by the system along the way. AI Clinician therefore ignores previous states of the system when making a decision and could potentially go against usual clinical practice by recommending sudden changes in the doses of vasopressor drugs.

AI Clinician is an assistive technology and so the whole clinical task is not delegated to the system. The final decision is made by the human clinician in charge of the patient’s care. Yet an important, cognitive part of the decision-making task is delegated: the interpretation of data. In transferring even part of the decision-making process to a machine, the control and epistemic conditions of responsibility are weakened.

The control condition is further compromised as follows. The complexity of the clinical setting is determined by the sepsis itself, the presence of factors such as new treatments, new diagnoses, new bacteria and viruses, as well as differences in patient care at earlier time points. It is difficult, however, to represent the clinical setting in the computational model during the design phase of the technology. Thus, the software’s behaviour may not fully reflect the clinical intentions on the system, since it was not feasible to specify them completely. This issue is currently dealt with by ignoring aspects of the process (for example, by limiting the number of inputs of information compared with those received by human clinicians). But there may be unintended consequences. For example, insensible losses of fluid cannot be electronically recorded, which could lead to the machine suggesting more fluids are needed when a clinician can see that the patient is already waterlogged. Furthermore, the machine may interpret the data in a way that does not reflect the human clinician’s reasoning as to what is most important in the context. For example, a clinician might choose to ignore a highly anomalous blood test result that could have been due to an error in blood sampling, test processing or result transcription. 

With respect to the epistemic condition, it is difficult to understand what constitutes best practice in the treatment of sepsis in hospitals, for several reasons. First, there are a variety of approaches used by clinicians to treat sepsis. Second, there can be differences in practice between clinicians (who set a general overview of the care plan) and nurses (who are responsible for minute-by-minute changes in care). Third, is the fact that best practice changes, both in terms of an evolving understanding of optimal treatment (for example, the move away from giving fluids towards use of vasopressor drugs) and in terms of new diseases and treatments that prompt questions about the meaning of optimal care. The epistemic condition is further compromised by two features of the machine itself: its own partial interpretation of the operating environment; and the opacity of many of its decisions even to the designers and users.

## Clinician and patient perspectives

An integral part of any complex health-care system is the implicit promise that clinicians and health-care organizations make to patients: to exercise good judgement, act with competence and provide healing.^22^ Moral accountability helps to avoid professional complacency and it underpins the patient’s trust in the clinician providing care. Patients tend to believe that the clinician is acting towards them with goodwill. However, goodwill is irrelevant, to the decisions reached by a software program. If human clinicians do not have robust control and knowledge of an artificial intelligence system’s recommendations, then it would be reasonable for a patient to want to know why those recommendations were followed.

We can describe the artificial intelligence system as only advisory and expect that accountability is thereby secure, because human clinicians still make the final decision. However, this action potentially results in a dilemma with two equally undesirable choices. Either clinicians must spend the time to develop their own opinions as to the best course of action, meaning that the artificial intelligence system adds little value; or clinicians must accept the advice blindly, further weakening both the control and epistemic conditions of moral accountability. The same dilemma affects safety assurance, as it becomes impossible to assure the system in isolation, because the system being assured now includes the clinician.

In the absence of a clinician’s direct, deliberate control over the recommendations reached by an artificial intelligence system, and given the opacity of many of these systems, safety assurance becomes increasingly important to both clinicians and patients. Safety assurance provides grounds for confidence that the patient safety risk associated with the system remains as low as reasonably possible. However, the static nature of most current safety cases does not cope with the dynamic characteristics of either clinical settings or machine learning. The adaptive behaviour of artificial intelligence systems typically alters the clinical environment, thereby invalidating the assumptions made in the safety case.^15^ In addition, the intended function of an artificial intelligence system is extremely diverse, and only partially understood by everyone involved, particularly the developers and the clinicians. The intended function cannot therefore be fully represented in the concrete specification that is used to build the system and which the system implements. The specification (in our example, sepsis treatment in intensive care) is based on a limited set of data points (vital signs and laboratory results). It is therefore much harder to assure, through a static safety case, that the system’s specified function preserves safety in all clinical scenarios in which it will operate. It becomes increasingly hard to assess the actual risk of harm to patients and this difficulty presents an epistemic challenge to the safety engineer. The difficulty of mitigating the actual risk of harm to patients presents a control challenge to the safety engineer.

From a technical engineering perspective, consideration of the safety of artificial intelligence is often limited to robustness: the properties of the system that might reduce its performance and availability, but not necessarily lead to patient harm.^23^ An example is model overfitting, where the predictive model fails to generalize beyond the set of data from which the model was derived. Technologists often fail to trace the impact of these technical properties on patient harm; for example, how biases in the artificial intelligence training data could compromise the safety of diagnosis in certain minority communities.

## The way forward

Determining where moral accountability lies in complex socio-technical systems is a difficult and imprecise task. One of the important current debates in patient safety is how to balance accountability across individual clinicians and the organizations in which they work.^24^^,^^25^ We argue for a need to include artificial intelligence developers and systems safety engineers in our assessments of moral accountability for patient harm. Meanwhile, none of the actors in the model robustly fulfil the traditional conditions of moral accountability for the decisions of an artificial intelligence system. We should therefore update our conception of moral responsibility in this context. We also believe in the need to move from a static to a dynamic model of assurance, accepting that considerations of safety are not fully resolvable during the design of the artificial intelligence system before the system has been deployed.^26^ This shift should include some consensus as to how much weakening of control, and what level of epistemic uncertainty is acceptably safe and morally justifiable before a digital system has been deployed, and in what context.

Moral accountability and safety assurance are continuing issues for complex artificial intelligence systems in critical health-care contexts. As such, it will be important to proactively collect data from, and experiences of, the use of such systems. We need to update safety risks based on actual clinical practice, by quantifying the morally relevant effects of reliance on artificial intelligence systems and determining how clinical practice has been influenced by the machine system itself. To do this we need an understanding of how clinicians, patients and the artificial intelligence systems themselves adapt their behaviour throughout the course of their interactions.
